# Use of Arthropod Rarity for Area Prioritisation: Insights from the Azorean Islands

**DOI:** 10.1371/journal.pone.0033995

**Published:** 2012-03-30

**Authors:** Simone Fattorini, Pedro Cardoso, François Rigal, Paulo A. V. Borges

**Affiliations:** 1 Azorean Biodiversity Group, Universidade dos Açores, Departamento de Ciências Agrárias CITA-A, Pico da Urze, Angra do Heroísmo, Portugal; 2 Water Ecology Team, Department of Biotechnology and Biosciences, University of Milano Bicocca, Piazza della Scienza 2, Milan, Italy; 3 Smithsonian Institution, National Museum of Natural History, Washington, D.C., United States of America; University of Western Ontario, Canada

## Abstract

We investigated the conservation concern of Azorean forest fragments and the entire Terceira Island surface using arthropod species vulnerability as defined by the Kattan index, which is based on species rarity. Species rarity was evaluated according to geographical distribution (endemic vs. non endemic species), habitat specialization (distribution across biotopes) and population size (individuals collected in standardized samples). Geographical rarity was considered at ‘global’ scale (species endemic to the Azorean islands) and ‘regional’ scale (single island endemics).

Measures of species vulnerability were combined into two indices of conservation concern for each forest fragment: (1) the Biodiversity Conservation Concern index, BCC, which reflects the average rarity score of the species present in a site, and (2) one proposed here and termed Biodiversity Conservation Weight, BCW, which reflects the sum of rarity scores of the same species assemblage. BCW was preferable to prioritise the areas with highest number of vulnerable species, whereas BCC helped the identification of areas with few, but highly threatened species due to a combination of different types of rarity.

A novel approach is introduced in which BCC and BCW indices were also adapted to deal with probabilities of occurrence instead of presence/absence data. The new probabilistic indices, termed pBCC and pBCW, were applied to Terceira Island for which we modelled species distributions to reconstruct species occurrence with different degree of probability also in areas from which data were not available. The application of the probabilistic indices revealed that some island sectors occupied by secondary vegetation, and hence not included in the current set of protected areas, may in fact host some rare species. This result suggests that protecting marginal non-natural areas which are however reservoirs of vulnerable species may also be important, especially when areas with well preserved primary habitats are scarce.

## Introduction

Protected areas are considered one of the most effective and cost-efficient ways to conserve habitats and viable populations of species, representative of the biological diversity of the Earth [1], [2]. However, also the landscape outside reserves could have an important, albeit usually overlooked, role in the conservation of particular species [3]–[6]. Selection of priority areas for biological conservation has been long driven by sociological, economical, and practical reasons, sometimes with tenuous scientific support [7], [8]. Recent developments in systematic conservation planning have put forward the need for more scientifically well-founded criteria for area prioritisation [7]–[13]. Obviously, one of the most commonly used criteria for locating areas of conservation concern is the presence of target species [14], [15], [16] or biotopes [17]. However, preserving umbrella or indicator species does not necessarily coincide with preserving the biodiversity at large and protected areas established for conserving certain target species do not protect automatically all imperilled species [18], [19]. The identification of biodiversity hotspots and the selection of priority areas are still generally based on the occurrence of target species among vertebrates and vascular plants [20]. This contrasts with the fact that invertebrates, and in particular arthropods, are the most diverse and abundant animal group in virtually all biotopes, performing a number of ecosystem functions that are irreplaceable [21], and include the vast majority of species threatened by extinction [22]. In general, it is assumed that invertebrates are too poorly known for driving conservation decisions [20]. This is due to a number of impediments, namely the scarce or non-existent knowledge about most species [21], including about their distribution (the Wallacean shortfall [23]), changes in space and time (the Prestonian shortfall [21]) and vulnerability to habitat change (the Hutchinsonian shortfall [24]).

A variety of species distribution modelling techniques [25] has recently been developed, and their application in conservation planning has been advocated [26], allowing a possible practical solution for the Wallacean shortfall [21]. In addition, recent work showed that it is relatively easy to obtain reliable measures of species threat status and conservation value for arthropods, similar to those used for plants and mammals [21], [27], [28], which proved important to partly overcome the Prestonian and Hutchinsonian shortfalls [21].

The Azorean Islands, a remote archipelago in the Atlantic Ocean, offer the unique opportunity of exploring the contribution that arthropods can offer to the identification of rarity hotspots. In these islands, the vast majority of endemic species are arthropods [29]. Yet, selection of priority areas for conservation on these islands has been mainly driven by biotopes, rare vascular plants and a few vertebrates [30], [31]. In this paper, we took advantage of data collected during a long term project of arthropod inventorying and monitoring [32]–[35], in order (1) to develop a multidimensional characterization of arthropod species rarity based on standardized sampling; (2) to use rarity measures to derive indices of species vulnerability to extinction; and (3) to use such indices to classify areas according to the vulnerability of the species they harbour. This was done for (1) all native forest fragments in all the Azorean islands and (2) all areas, irrespectively of their biotope type, in Terceira, the island with the most comprehensive data.

## Methods

We employed a multistep modelling approach to identify priority areas for conservation. In the following sections, we describe the main points of our methodological framework. Further details about the analyses are given in Information S1.

### Study areas and sampling

The Azores archipelago stretches out over 615 km in the North Atlantic Ocean (37–40°N, 25–31°W), 1584 km west of southern Europe and 2150 km east of the North American continent. The native forest in the Azores is characterized by an association of native (many endemic) evergreen shrub and tree species. Commonly known as Laurisilva, this forest occupied most of the surface of all the islands before human settlement almost 600 years ago. However, native forests are now mostly restricted to high and steep areas, while most of the islands are covered by exotic plantations of *Cryptomeria japonica* and *Eucalyptus* spp., abandoned fields now dominated by *Pittosporum undulatum*, semi-natural pastures, and intensively managed pastures. Although protected native forest covers less than 3% of the total area of the archipelago, it is the biotope in which the great majority of the endemic plant and animal species occur in the Azores [32], [33], [35], [36].

In this study, we first considered 18 native forest fragments distributed across seven islands of the archipelago: Santa Maria, São Miguel, Terceira, São Jorge, Faial, Pico and Flores (Table 1, see [36] for details). This corresponds to most of the native forest extent of the Azores. All these areas are now protected under different regimes [37].

**Table 1:** IUCN levels of protection (according to [37]), Index of Biodiversity Conservation Concern, and Index of Biodiversity Conservation Weight for 18 native forest fragments on the Azorean Islands.

Forest Fragment (island)	IUCN levels of protection	BCC with SIE	BCC with AZE	BCW with SIE	BCW with AZE
Atalhada (S. Miguel)	IV	0.132	0.329	0.145	0.248
Biscoito da Ferraria (Terceira)	I	0.153	0.375	0.186	0.311
Cabeço do Fogo (Faial)	IV	0.111	0.259	0.094	0.153
Caldeira do Faial (Faial)	I	0.122	0.332	0.096	0.181
Caldeira Guilherme Moniz (Terceira)	VI	0.094	0.252	0.091	0.167
Caldeiras Funda e Rasa (Flores)	I	0.111	0.329	0.113	0.231
Caveiro (Pico)	I	0.110	0.350	0.108	0.237
Graminhais (S. Miguel)	IV	0.090	0.329	0.071	0.179
Lagoa do Caiado (Pico)	IV	0.094	0.337	0.086	0.212
Mistério da Prainha (Pico)	I	0.094	0.300	0.127	0.276
Morro Alto e Pico da Sé (Flores)	I	0.132	0.335	0.148	0.256
Pico Alto (Sta Maria)	IV	0.155	0.348	0.185	0.284
Pico da Vara (S. Miguel)	I	0.119	0.305	0.147	0.257
Pico do Galhardo (Terceira)	IV	0.102	0.316	0.112	0.238
Pico Pinheiro (S. Jorge)	IV	0.129	0.350	0.137	0.255
Serra Sta. Bárbara (Terceira)	I	0.157	0.374	0.219	0.354
Terra Brava (Terceira)	I	0.110	0.326	0.138	0.279
Topo (S. Jorge)	V	0.128	0.385	0.124	0.256

IUCN levels of protection: I –Natural Reserve; III –Natural Monument; IV –Habitat and species management; V –Protected Landscape; VI –Resources management.

BCC: Index of Biodiversity Conservation Concern; BCW: Index of Biodiversity Conservation Weight.

BCC and BCW were calculated using single island endemics (SIEs) and Azorean endemics (AZEs).

In each forest fragment, arthropod sampling was conducted using the same standardized protocols to collect both ground dwelling arthropods (by pitfall traps) and canopy arthropods (by beating). Using the same sampling protocol we also collected individuals across six different land uses (i.e. high altitude natural grasslands, peat bogs, exotic forests, semi-natural pastures, intensively managed pastures, canopies of orchards) for six islands: Santa Maria, Terceira, São Jorge, Faial, Pico, and Flores (see [33], [38], [39] and Information S1).

All necessary permits from the Azorean Nature Parks for each of the studied island were obtained for the described field studies. None of the species sampled are protected by Azorean, Portuguese or International laws. However, this sampling allowed us to inform the Azorean Government about the distribution of restricted endemic species for improving the design of current Protected Areas (Borges et al., unpublished Reports).

### Measures of species rarity

In order to fulfil Hartley and Kunin's recommendations of considering different aspects of rarity [40], species rarity was assessed here using a multidimensional characterization that takes into account: (1) geographical distribution (wide/narrow distribution), (2) abundance (abundant/scarce population), and (3) habitat specificity (low/high habitat specificity) [41], [42]. Such a multidimensional characterization of species rarity has been successfully applied to vertebrates [43], [44], [45], arthropods [46]–[49] and bryophytes [50].

### Geographical distribution

Estimating the geographical rarity of a species depends on the spatial scale of analysis [51], [52], so we adopted a two-level approach. At a global level, we considered as geographically rare the species which are endemic to the Azorean Islands, even if distributed in more than one island (hereafter AZE species). At a regional level, we considered as geographically rare the species which are endemic to single Azorean Islands (singe island endemics, hereafter SIEs). Endemics are typically considered as taxa of conservation concern [53], [54], and this approach also ensures that endemic taxa are scored as important, at least in terms of geographical rarity, from a global and a regional perspective.

### Abundance

To calculate the relative abundance of each species in the Azores we used all the standardized transects available for all main biotopes in seven of the nine islands (Corvo and Graciosa were not sampled since they have entirely lost their native forest; see more details in [33], [35], [39], and Information S1). Species with abundance below the median were classified as rare.

### Habitat specificity

We used species abundances across the biotopes occurring on the study islands to calculate species habitat specificity using the Shannon *H*′ index [55]. Species with *H*′ values below the median were classified as rare [43].

### Vulnerability index

Species with smaller ranges, lower abundances and narrower biotope ranges tend to experience higher levels of threat [45]. Thus, using species categorisation into the rarity forms described above (i.e. geographic distribution, abundance and habitat specificity), we calculated an index of species vulnerability as proposed by Kattan [43].

We calculated two measures of the Kattan index, considering alternatively as geographically rare only SIEs or all the AZEs. *χ* ^2^- tests were used to determine the independence of the three measures of rarity [43].

Spearman rank correlations were used to test inter-correlations among number of islands from which a species is known (NISL), number of biotopes occupied by a species (NBIO), *H*′ measure of habitat specificity, species abundance, and Kattan indices. NISL and NBIO were considered as measures of geographical rarity and habitat specificity alternative to those used to construct the Kattan index. Correlations between the Kattan index and these two measures indicate that the index is robust to different ways of calculating species rarity.

### Forest fragment ranking

We ranked forest fragments according to two different measures of prioritisation.

- We used the Biodiversity Conservation Concern (BCC) index [56] whose original formulation was modified to make it more general, as observed in [57]. With the new formulation, BCC can be calculated as:
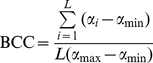
(1)where *L* is the local species richness, *α_i_* is the vulnerability index assigned to the *i*th species (as defined above), *α*_min_ is the minimum weight among all species; and *α*_max_ is maximum weight among all species.

The BCC index has been previously applied to identify priority areas or biotopes for butterflies in Mediterranean islands and European countries [56]–[59], fish in France [60], tenebrionids, butterflies, birds and mammals in the Central Apennines [47], [48].

- We also used a new index, the Biodiversity Conservation Weight (BCW) index, also based on species vulnerability. The BCC index is a ‘relative measure’, which means that it is not sensitive to species richness. This may be an advantage to compare species assemblages with different species richness [48], [56], but poses some problems. For example, an assemblage with a single species, having this species *α*_max_, would receive the same score as an assemblage with 10 species, all with *α*_max_. Or worse, an assemblage with a single species with *α*_max_ has a higher score than an assemblage with 10 species, 9 with *α*_max_ and one with *α*_i_<*α*_max_. To overcome this problem, we have calculated the BCW as follows:
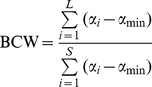
(2)where *S* is the total species richness for all sites (all other symbols as for BCC, see above).

Spearman rank correlations were used to test inter-correlations between BCC and BCW values.

### Potential distribution modelling

In many cases the features to rank are not discrete, relatively large, units for which most existing species are known, such as the 18 Azorean forest fragments. Especially for arthropods and other small organisms, just because a species is not known from a site does not mean it is not present. Often it was just not searched for or not found and such site can be overlooked in conservation priority exercises.

Thus, for Terceira, the island for which more information was available, we calculated and mapped potential BCC and BCW (pBCC and pBCW) based on probabilistic species distributions. For this, we used the maximum entropy algorithm [61], [62] to model species distributions on this island using climatic data, landscape maps and topographical and geographical information [63]–[65] (see Information S1 for details).

### Mapping of potential rarity

The BCC and BCW indices were designed to deal with occurrence data, not with probabilities of occurrence. One possibility to use them with the latter type of data would be to convert probability maps into presence/absence maps by using a threshold in probabilities above which the species would be considered to be present [66]. This would however cause three shortcomings. Firstly, the best threshold is hard to define, although a few guidelines exist [66], [67]. Secondly, this would imply a loss of information. Thirdly, this would consider as completely different some sites with very similar species composition if such sites were very close to the threshold for one or a few rare species.

Thus we preferred to use modified versions of the BCC and BCW formulas to explicitly cope with probabilities of occurrence (see Supplementary Information about Methods for details). The formulas for potential BCC (pBCC) and potential BCW (pBCW) are therefore:
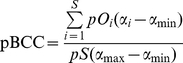
(3)and
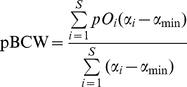
(4)where for each cell: *S* is the total species richness for all sites; *pS* is the potential species richness (*pS* = 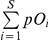), *pO_i_* is the probability of occurrence of species *i*, α*_i_* is the weight of species *i*; α_min_ is the minimum weight among all species and α_max_ is the maximum weight among all species.

## Results

In total, we considered 219 arthropod species, 178 of which are found in the 18 studied protected areas. Of these 178 species, 82 are considered Azorean endemics (AZE) and of those 26 are Single Island Endemics (SIEs) (see Information S2).

### Vulnerability index

**Figure 1:**
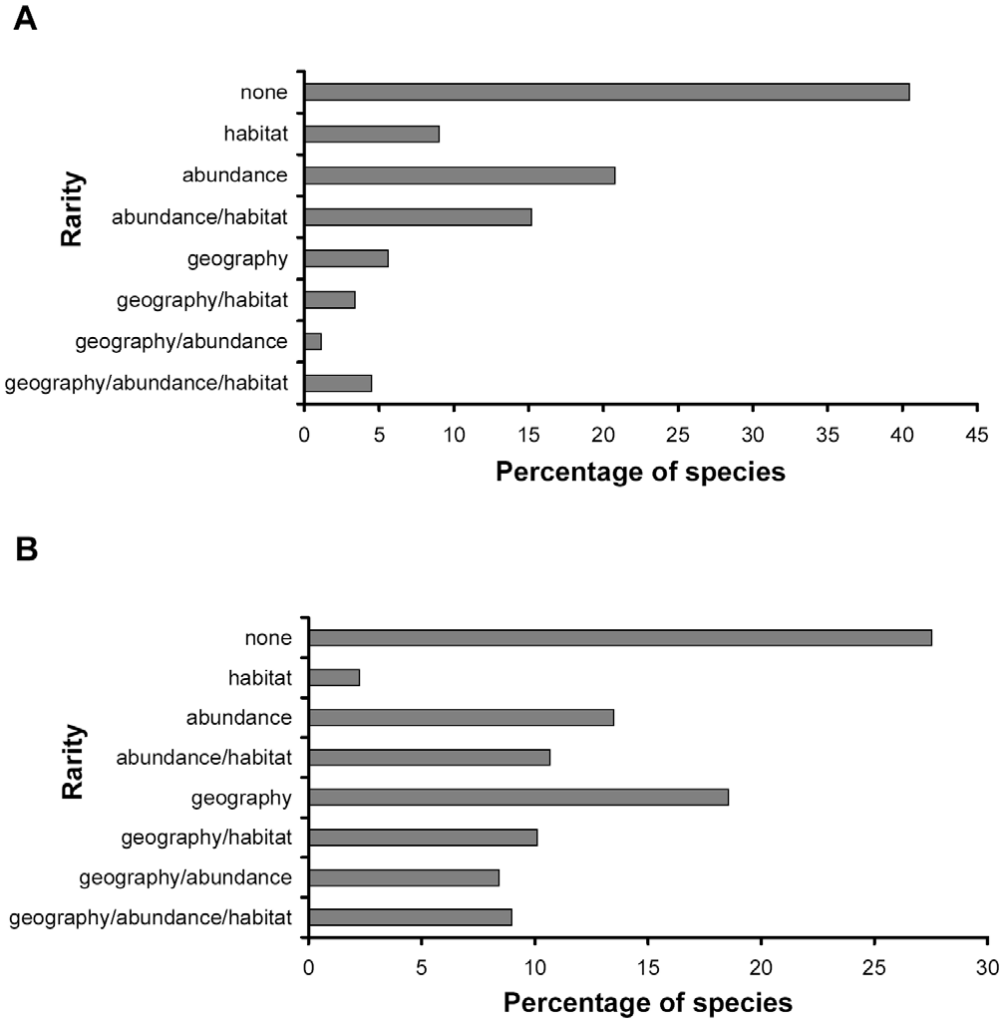
Percentages of the seven categories of arthropod rarity. A total of 178 arthropod species in 18 forest fragments in the Azorean Islands were considered with different criteria for endemics: (**A**) only single island endemics (SIEs) were considered geographically rare; (**B**) all Azorean endemics (AZEs) were considered geographically rare.

Although non-rare species were the most abundant category (28–40% according to the measure of geographical rarity which is used), a high proportion of species was rare for at least one criterion (Fig. 1). Using the SIE criterion, about 5% of the species were rare for all rarity dimensions (geography, abundance and habitat). This percentage increased substantially with the use of AZEs reaching close to 10%.

The results of the *χ* ^2^ tests indicate that the hypothesis of overall independence of the three rarity dimensions is rejected (Information S3). However, separate analyses of the 2×2 tables indicate that distribution and abundance are jointly independent factors (Information S3).

Both Kattan indices were strongly correlated with the original measures of species habitat specialization (*H*′) and abundances from which the indexes have been obtained (Information S4). Interestingly, both Kattan indices were also correlated with the number of biotopes a species occupies and the number of islands from which a species is known, which can be considered alternative measures of habitat specialization and geographical rarity (Information S4).

### Forest fragment ranking

Values of Index of Biodiversity Conservation Concern (BCC) and Index of Biodiversity Conservation Weight (BCW) are reported in Table 1, and their intercorrelations in Information S5.

Although correlation values between indexes varied, the following fragments were consistently placed in the third quartiles for all four indices (BCC and BCW using SIEs and AZEs): Serra Sta. Bárbara, Biscoito da Ferraria (the two largest fragments in Terceira) and Pico Alto (the only fragment in the oldest island, Santa Maria).

Focusing on the top five ranked fragments (third quartile) for each index (Table 2), the five fragments selected by the BCC and BCW with SIEs captured about 80% of the entire species richness of all 18 fragments. Species captured by these two indices showed also relatively high mean values for vulnerability indices (Table 2).

**Table 2:** Number (and percentages) of species included in the first five ranked fragments according to Index of Biodiversity Conservation Concern and Index of Biodiversity Conservation Weight, with indication of Mean (and Standard Deviation) values of vulnerability (Kattan index) of the species included in the selected fragments.

	BCC with SIE	BCC with AZE	BCW with SIE	BCW with AZE
Captured species richness (%)	141 (79.2)	115 (64.6)	139 (78.1)	130 (73.0)
Mean (SD) value of Kattan index of included species with SIE criterion	2.454 (1.830)	2.409 (1.910)	2.511 (1.931)	2.377 (1.814)
Mean (SD) value of Kattan index of included species with AZE criterion	3.624 (2.316)	3.765 (2.313)	3.698 (2.370)	3.638 (2.353)

BCC: Index of Biodiversity Conservation Concern; BCW: Index of Biodiversity Conservation Weight. BCC and BCW were calculated using single island endemics (SIEs) and Azorean endemics (AZEs) as alternative criteria for geographical rarity.

### Mapping of potential rarity

**Figure 2:**
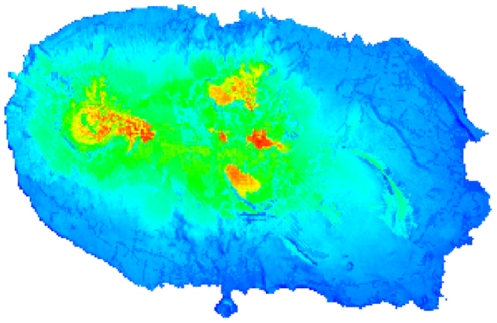
Potential arthropod species richness on Terceira Island. Species richness is based on probability of occurrence. Colder colours (dark blue) represent low values (minimum value = 4.055) and hot colours (red) represent high values (maximum value = 29.251). The theoretical range is 0–47 as 47 species were evaluated.

All 47 species probability maps had AUC values above 0.7 and we considered them as reliable (Information S6). Highest values of potential species richness (Fig. 2) were concentrated in the five forest fragments of Terceira: Serra Sta. Bárbara, Biscoito da Ferraria, Terra Brava, Pico Galhardo and Caldeira de Guilherme Moniz.

**Figure 3:**
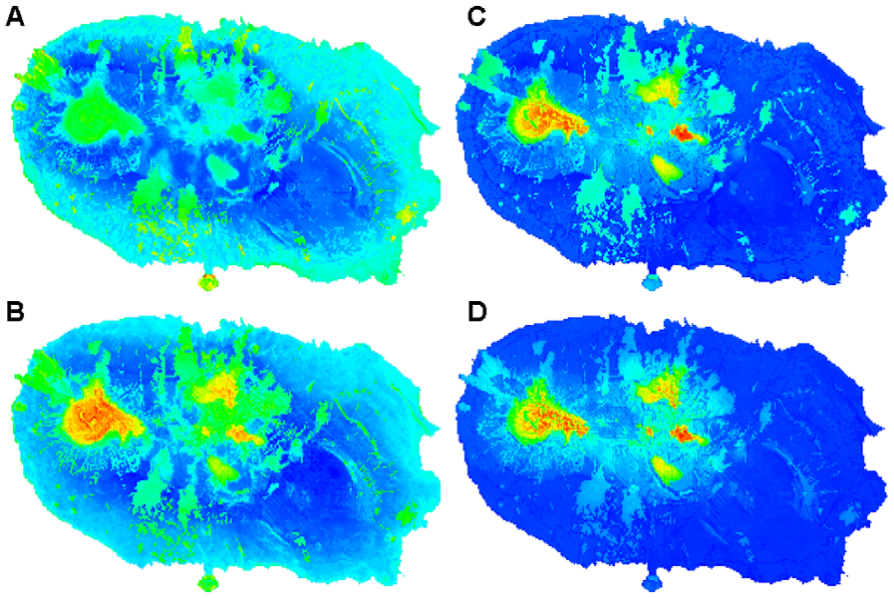
Maps of indices of arthropod conservation in Terceira. **A** and **B** illustrate potential Biodiversity Conservation Concern (pBCC). **C** and **D** illustrate potential Biodiversity Conservation Weight (pBCW). Colder colours represent low values and hot colours represent high values. Maps of figures **A** and **C** were calculated using only single island endemics (SIEs) as geographically rare species (ranges: 0.031–0.175 and 0.072–0.553, respectively). Maps of figures **B** and **D** were calculated using all Azorean endemics (AZEs) as geographically rare species (ranges: 0.081–0.282 and 0.051–0.638, respectively).

Use of pBCC with Azorean endemics produced a somewhat similar pattern (Fig. 3B), while the use of only SIEs as geographically rare species highlighted a more complex pattern (Fig. 3A). This more restrictive SIE approach, more than for the aforementioned areas, gave relatively high scores to the protected areas of Monte Brasil (southernmost tip of the island) and Serreta (northeastern Terceira), in the coastal areas of the island. The pBCC with SIE also highlighted an important patch in the southwestern part of the island (Fonte do Bastardo). Use of pBCW (Fig. 3 C and D) gave results somewhat similar to those achieved using potential species richness or pBCC with Azorean endemics, although even more strongly emphasizing the importance of native forest fragments.

**Figure 4:**
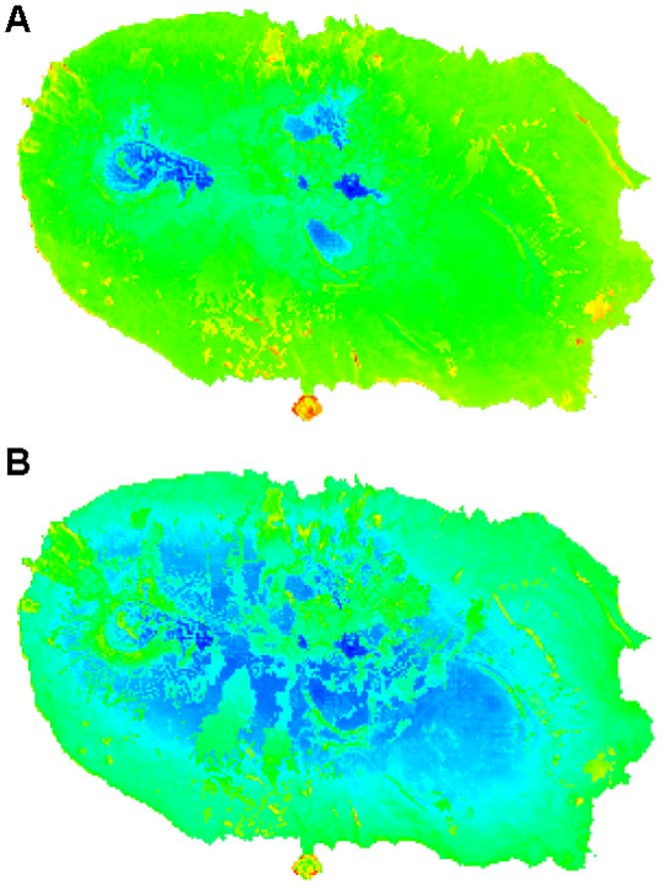
Differences between potential Biodiversity Conservation Concern (pBCC) and potential Biodiversity Conservation Weight (pBCW). All maps were rescaled from 0 to 1 and pBCW were subtracted from pBCC. Cold colours represent sites where pBCW is higher than pBCC and hot colours represent sites where pBCC is higher than pBCW. Values were calculated using only single island endemics (SIEs) (**A**) and all Azorean endemics (AZEs) (**B**) as geographically rare species.

To emphasize differences in the outputs of pBCC and pBCW, we rescaled previous maps from 0 to 1 and did a simple subtraction of pBCC from pBCW (Fig. 4). This shows that pBCC is giving more importance to low altitude areas, most notably Monte Brasil, while pBCW is giving more importance to native forests or high altitude areas.

## Discussion

### Rabinowitz's approach to rarity

Previous studies using Rabinowitz's forms of rarity [41], [42] found that while a high proportion of species have relatively small geographical ranges, only few species are widespread and abundant, and the condition of ‘abundant and localized’ is extremely rare since locally abundant populations tend to rapidly occupy new sites [51], [52], [68], [69]. However, it is noteworthy to consider the scale of analysis, and hence the way geographical rarity is assessed. When considering as geographically rare only the SIEs, we found a relatively small percentage (about 9%) of species which were abundant and geographically restricted. But this percentage was about 29% when endemics were considered as geographically restricted. That is, almost one third of the AZEs at the archipelago scale were considered abundant, which implies that many of the endemics that were able to occupy more than one island were also successful in building large populations in most islands. Because the Kattan index used as a vulnerability measure in the BCC and BCW indexes gives more weight to geographical rarity, it is critical to carefully consider the scale of analysis.

Moreover, comparisons of multiple taxa within the same geographical context revealed that proportions of different categories of rarity tend to change considerably among taxa [48]. Thus, no generalization seems possible and rarity measures always have a relative value, depending on the particular assemblage of species under study (cf. also [43], [44], [45]).

On the other hand, the Kattan index was very efficient in summarizing the three dimensions of rarity and it was also proven to be robust to variations in the way geographical rarity and habitat specificity is measured. This qualifies the Kattan index as a good synthetic measure of species ‘rarity’.

### Prioritisations of biotopes and areas (BCC vs. BCW)

Although species are the primary target of conservation efforts, a number of impediments, including the Linnean shortfall (incomplete taxonomic knowledge), the Wallacean shortfall (incomplete information on species distribution), the Prestonian shortfall (lack of adequate estimates of population abundance and changes in space and time) and the Hutchinsonian shortfall (incomplete knowledge of species relationships with the environment) [21] make generally impractical the adoption of species-focused actions (e.g. action plans) for arthropods. Thus, arthropod conservation is generally based mainly on the identification of priority sites selected by the occurrence of priority species [70], assuming that preservation of the biotope of that/those species will automatically allow conservation of other imperilled species [71], [72]. Rarity measures are widely recognized as good surrogates of species extinction risk and can be obtained also when information on species taxonomy, distribution, population size and biology is limited, thus surpassing the aforementioned shortfalls. Also, their combined use in the Kattan index may be particularly useful to obtain a general evaluation of species vulnerability. After a large number of species are evaluated, their vulnerability can be used to identify priority areas.

In this study, we used two indices based on species vulnerability, the Biodiversity Conservation Concern (BCC, introduced by [56]) and the Biodiversity Conservation Weight (BCW) (introduced here) to prioritise forest fragments. The results provided by these two indices generally differ. BCC places more emphasis on species-poor areas which may contain, however, high proportions of mostly vulnerable species, whereas BCW tends to identify areas which have large numbers of highly vulnerable species. Although the BCW may appear to give a more logical signal, BCC can be used to drive attention to areas with few, but very rare threatened species. This can be important for areas occupied by biotopes which host few, but highly specialized species, such as high altitude open biotopes [48] or caves [73]. For the best preserved areas, the two indices tend to give similar prioritisations, but the BCC tends to emphasize degraded areas which still host few imperilled species. This calls attention for the need to create additional measures of conservation management to non-natural areas [3], [4], [5]. In small territories like islands in which the matrix surrounding the protected areas concentrates most of the intensive forest and agriculture activities, those species located in isolated pockets are in high danger of extinction.

It is noteworthy that the BCC and BCW indices tend to give the highest values to the same fragments when using different criteria of geographical rarity. However, the two indices may give different results in less obvious cases, for example for fragments with few, but very vulnerable species. An important source of bias in the use of these indices in locating priority areas may be the inadequate knowledge of species distribution (Wallacean shortfall). In particular, failure to detect species in areas where they are in fact present, can bias results in favour of the best sampled areas. For example, comparing known patterns of Amazon plant diversity with those reconstructed using modelled full distributions, Hopkins [74] showed that the ‘real’ diversity map of Amazonian plant richness might be very different from the ‘known’ pattern. For this reason, in our study, we modelled potential arthropod species distribution on Terceira Island, and then calculated for each geographical unit the pBCC and pBCW indices on the basis of the probability of occurrence of each species. This novel approach allowed the identification of some areas that are potentially important for the conservation of biodiversity in Terceira Island, even if such areas were never sampled. For example, the area of Monte Brasil, not included – and hence not evaluated – among the analysed forest fragments because occupied by secondary vegetation, may also be important to preserve if the objective is to guarantee the persistence of the endemic biota. Some endemic species (in particular low altitude specialized species, such as the endemic weevil *Drouetius azoricus parallelirostris*) still occur in this area.

### Patterns of prioritisation for the Azorean native forest fragments

We have previously examined the relative value of 18 forest fragments in seven of the Azorean islands to improve the conservation of Azorean soil epigean arthropod biodiversity [32], [36]. In this current contribution, we evaluate the ability of different indices to reflect species assemblage importance, calculating the percentage of total richness included in the top ranked fragments for each metric (see also [75]). On the whole, the top five fragments included about 65–80% of total richness. The best results were obtained using BCC and BCW with SIEs. Thus, the use of SIEs seems to select areas which capture more species than those found using AZEs. This highlights the importance of native fragments that have unique species like the small and disturbed area of Pico Alto in Santa Maria (see also [32], [39]).

When ranking sites based on BCW, top native protected areas are mainly large pristine reserves, with exception of Pico Alto in Santa Maria. Pico Alto region is located in the archipelago's oldest island and is a hotspot of biodiversity [32], in which over 57 endemic arthropod species are known, *i.e.* 21% of the Azorean endemic arthropods occur in an area representing <0.25% of Azorean native forests.

IUCN levels of protection for the Azorean native forests not always gave the higher priority to the most important areas. This is the case of Pico Alto (Santa Maria), Atalhada (São Miguel) and Pico Pinheiro and Topo (São Jorge) that score high in BCC – SIE or BCC – AZE, but have only a level of protection IV or V in the Azores (see Table 1). Most of these areas are highly disturbed [36], but still maintain important populations of unique species. This reveals the importance of considering not only a dual classification of protected/unprotected in spatial conservation planning, but to consider also the category of the protected areas and how well each category is able to guarantee the persistence of each species in the future. If some species are able to withstand some human intervention over their habitat, other may not and low protection categories may be insufficient.

### Conclusions

We used two indices to rank Azorean forest fragments and the entire area of Terceira Island according to arthropod species vulnerability. To assess species vulnerability we referred to species rarity. Species rarity was evaluated according to geographical distribution, habitat specialization and population size of the species. Because geographical rarity can be assessed at different scales, we performed our analyses considering two possible classifications: at ‘global’ scale, we considered as rare the species endemic to the Azorean islands (AZEs); at ‘regional’ scale, only those endemic to single islands (SIEs). These alternative measures of geographical rarity tend to produce different outcomes. We think no particular choice can be recommended in general, because it depends on the aim of the study. In our case, for example, the use of SIEs may be more appropriate to prioritise forest fragments among islands because it enhances the total number of species included in the final set of prioritised areas. Using synthetic indices to prioritise areas according to species vulnerability also raises the problem whether applying an absolute or a relative measure, i.e. whether considering the overall weight obtained by the sum of the vulnerability measures of the species occurring in a given area (as in the BCW), or dividing this sum by species richness (as in the BCC). In general, an absolute index seems preferable to prioritise the areas with the highest numbers of vulnerable species, but a relative index may help the identification of areas with few, but highly imperilled species. Thus, the two approaches should be used in tandem for a ‘balanced’ overview of conservation priorities. Because areas are ranked on the basis of the species they host, incomplete knowledge of species distributions can produce wrong prioritisations in favour of the best sampled areas. Moreover, common practice to rank areas in biological conservation is to define a priori the areas to compare and then to rank them according to the species. This might overlook important areas which where not considered because of lack of data. Recent development of procedures to model species distributions allows the reconstruction of maps of species occurrence with different degrees of probability covering areas from which data are not available. An application of such approach to the arthropods of Terceira revealed that some island sectors occupied by secondary vegetation, and hence not included among the areas analysed for forest fragment prioritisation, may in fact host some vulnerable species. The natural landscapes of the Azorean Islands have been almost completely destroyed and primary forests are reduced to very few, sparse and small fragments. In such circumstance, protecting non-natural areas which are however reservoirs of imperilled species may be also important [3], [4], [5].

## Supporting Information

Information S1 **Detailed description of sampling procedures and statistical calculation.**
(PDF)

Information S2 **Endemic status, number of occupied islands, number of occupied habitats, Shannon** ***H*** **′ index of habitat specificity and abundance of each arthropod species found in the Azorean forest fragments.**
(XLS)

Information S3 **Results of the χ^2^ tests of independence of the three dimensions of rarity for the Azorean arthropods.**
(PDF)

Information S4 **Correlation (Spearman rank coefficient) between measures of rarity for 178 arthropods of the Azorean Islands.**
(PDF)

Information S5 **Correlation (Spearman rank coefficient) between Index of Biodiversity Conservation Concern and Index of Biodiversity Conservation Weight for 18 Azorean forest fragments.**
(PDF)

Information S6 **Area Under the Curve (AUC) values for the potential distribution modelling of all 47 studied species.**
(PDF)
