# Supplementary material for: Use of Arthropod Rarity for Area Prioritisation: Insights from the Azorean Islands
Source: PLoS One. 2012 Mar 30;7(3):e33995. doi: 10.1371/journal.pone.0033995 (PMC3316514; doi:10.1371/journal.pone.0033995)
Supplement: Information S1 — Detailed description of sampling procedures and statistical calculation. (PDF) [file pone.0033995.s001.pdf]

Supporting Information S1

Detailed description of sampling procedures and statistical calculations

Study area and sampling

The Azorean archipelago includes nine main islands of recent volcanic origin, which are distributed in three groups: the Western group of Corvo and Flores; the central group of Faial, Pico, Graciosa, São Jorge, and Terceira; and the Eastern group of São Miguel and Santa Maria. The Azorean Laurisilva is distinguished from other Laurisilva forests of the Macaronesia by a dense tree and shrub cover of small stature (trees have an average height of 3 m), a closed canopy, high levels of humidity and scarce understorey light.

Currently, the original Azorean Laurisilva forest is restricted to scattered fragments because most of the islands are occupied by exotic plantations of *Cryptomeria japonica* and *Eucalyptus* spp., abandoned fields covered by *Pittosporum undulatum*, semi-natural pastures, and intensively managed pastures. Also important are a number of abandoned fields with exotic plants, agriculture fields, coastal biotopes (many highly disturbed), urban areas, recent lava-flows covered with the endemic early successional tree-shrub *Erica azorica* and volcanic lava-tubes and pits.

Nine natural parks encompassing a number of protected areas each have been recently established on the Azorean Islands, one in each island, based on the IUCN Management Categories System [37]. These preserves have been designed to fulfil different aims (including leisure), and the applied criteria usually included the preservation of particular vascular plants, vertebrates or biotopes, mainly native forest fragments. Invertebrates were only considered in a few cases of small forest fragments that no longer included other important taxa.

In this study, we sampled arthropods in 18 native Laurisilva forest fragments distributed across seven islands of the archipelago (see [36] for details). The remaining two islands (Corvo and Graciosa) currently have no native forest. In each forest fragment, we laid out a minimum of four independent 150-m long, 5-m wide transects in each fragment, with more transects available in

larger fragments (see [38]). We sampled the epigean arthropod fauna using 30 pitfall traps (100 ml each) per transect for at least a two-week period during summer months. Half of the pitfall traps contained an attractive solution (Turquin), while the remaining had a non-attractive solution with a small proportion of ethylene glycol. The two types of traps were placed alternately. Canopy arthropods from woody shrub and tree species were sampled using a beating tray in the same period of trap functioning. Ten beating samples were taken at 15m intervals from each of the three most dominant woody plant species in the site. For more details regarding this survey see [32] and [38].

In Santa Maria, Terceira, São Jorge, Pico, Faial and Flores Islands we performed additional sampling in other landuse types (see e.g. [39] for Santa Maria). Using 30 pitfall traps with both Turquin and ethylene glycol, additional transects were placed in each of five biotopes besides native forests: high altitude natural grasslands (20 sites in five islands), peat bogs (4 sites in one island), exotic forests (37 sites in four islands), semi-natural pastures (29 sites in four islands), and intensively managed pastures (38 sites in four islands) (see also [35]). Additionally, we used also data from the canopies of orchards (n= 58) from Terceira.

All arthropods were initially sorted into morphospecies by a team of trained persons and later identified to species (with few exceptions) by expert taxonomists.

Because most arthropod species in the Azores are exotic (60% of the arthropods [32]) and exotic species usually have no conservation value, we restricted all analyses to indigenous species only. Indigenous species refers to the species occurring only in the Azores (i.e. archipelagic endemics) or those that are considered to have arrived by long-distance dispersal to the Azores rather than by intentional or accidental human introduction, and also known from other regions (i.e. native species). Non-indigenous species are those believed to have arrived in the archipelago because of human activities and which often have a cosmopolitan distribution.

Calculation of species abundance

To calculate the relative abundance of each species, in addition to the transects in native Laurisilva forests (n= 135), we also considered transects in the following other biotopes: exotic forest (n= 37), orchards (n= 58), high altitude natural grasslands (n= 20), peat bogs (n= 4), semi-natural pasture (n= 29), and intensively managed pastures (n=38), if available (see more details in [33,35,39]. First, the mean abundance in each transect was calculated for each of the islands, and, secondly, the mean abundance of each species across the seven islands was calculated to obtain an overall abundance. Then we calculated the median value across these values of 'species overall abundances', and species below the median were considered as rare (see Supporting Information S2).

Calculation of habitat specificity

The mean abundance in each transect was calculated for each of the seven biotopes mentioned above. Then we used species abundances across the seven biotopes to calculate species habitat specificity using the Shannon index:

$$H' = -\sum \frac{A_i}{A} \ln\left(\frac{A_i}{A}\right).$$

where A_i is the mean abundance of the species in the biotope *i*, and *A* is the total abundance of that species across the biotopes (see [55]). Species with high habitat specificity tend to have lower *H'* values (with species restricted to a single biotope receiving *H'*=0), whereas species with low habitat specificity (i.e. which occur with similar population densities across a variety of biotopes) have large *H'* values (see Supporting Information S2).

Vulnerability index

To calculate Kattan values of species vulnerability we used the following procedure.

The three rarity forms yield a total number of eight possible combinations, ranging from most 'common' (vulnerability index 1: wide geographical distribution-high abundance-low habitat specificity) to 'rare' (vulnerability index 8: narrow distribution-low abundance-high habitat specificity) in all three dimensions. Of the remaining six cells, three represent rare species in two dimensions and three in a single dimension (vulnerability index values 2–7). To rank these cells, we considered that species with narrow geographical distribution are more vulnerable at a global/regional level, and species with high habitat specificity are more vulnerable regardless of their abundance. This led to the following categorisation: 1: common species; 2: low abundance species; 3: species with high habitat specificity; 4: geographically restricted species; 5: scarce species with high habitat specificity; 6: low abundance and geographically restricted species; 7: geographically restricted and high habitat specificity species; 8: low abundance species with restricted ranges and high habitat specificity. To determine the independence of the three measures of rarity [43], an overall 2 × 4 contingency table was preliminarily constructed with number of low/high abundance species in rows, and number of geographically restricted – habitat specialised/ non-specialised species and number of geographically widespread – habitat specialised/ non-specialised species in columns. Because examination of frequencies revealed that some combinations were particularly important in determining an overall significant or non significant result with a χ^2- test, the original table was subdivided into 2 × 2 tables and χ^2- tests with Yates correction for contiguity were performed.

Potential distribution modelling

We applied a maximum entropy algorithm [61,62] to model distributions of 47 arthropod species on Terceira. All species presences in 52 sites (13 in native forest, 13 in exotic forest, 13 in semi-natural pasture and 13 in intensively managed pasture) were used for distribution modelling. We only used pitfall trap data as these were the only available for all sites. The following environmental and geographical variables with a resolution of 100m were used for modelling:

- Climatic data extracted from [63] based on the CIELO Model for Azores [64]: (1) average annual temperature, (2) annual temperature range, (3) annual precipitation, (4) annual precipitation range, (5) annual radiation, (6) annual potential evapotranspiration;
- Landuse map (the only categorical variable) (7): was a result of aerial photography and fieldwork (F. Dinis, unpubl.) with additional information from [65].
- Topographic and geographic data: (8) altitude, (9) slope, (10) distance to the sea, (11) latitude and (12) longitude.
- Landscape data: (13) landscape disturbance index (Cardoso & Rigal, unpublished data).

Using a large number of variables in species distribution modelling may result in over-fitting with the consequence that the potential distribution of each species may be underestimated. Although this may be a problem in some applications, in spatial conservation prioritization it is indeed an advantage. In our case, as in similar works, commission errors, i.e., predicting a species to be present where it is in fact absent, are more serious than omission errors, i.e., predicting a species to be absent where it is in fact present. A high rate of commission may lead to a smaller number of areas to be suggested as necessary to encompass all species at appropriate levels for their protection.
