# Supplementary material for: Use of Arthropod Rarity for Area Prioritisation: Insights from the Azorean Islands
Source: PLoS One. 2012 Mar 30;7(3):e33995. doi: 10.1371/journal.pone.0033995 (PMC3316514; doi:10.1371/journal.pone.0033995)
Supplement: Information S3 — Results of the χ2 tests of independence of the three dimensions of rarity for the Azorean arthropods. (PDF) [file pone.0033995.s003.pdf]

Supporting Information S3 Results of the χ^2 tests of independence of the three dimensions of rarity for the Azorean arthropods.

	SIE			AZE		
**Test**	χ^2	**df**	***P***	χ^2	**df**	***P***
Geographic distribution × biotope × abundance	15.043	3	0.0016	20.745	3	0.0001
Distribution × abundance	0.018	1	0.894	0.624	1	0.429
Abundance × biotope	12.401	1	0.0004	12.401	1	0.0004
Biotope × distribution	5.539	1	0.019	5.447	1	0.020

SIE: single island endemics; AZE: Azorean endemics.
