# Supplementary material for: Use of Arthropod Rarity for Area Prioritisation: Insights from the Azorean Islands
Source: PLoS One. 2012 Mar 30;7(3):e33995. doi: 10.1371/journal.pone.0033995 (PMC3316514; doi:10.1371/journal.pone.0033995)
Supplement: Information S4 — Correlation (Spearman rank coefficient) between measures of rarity for 178 arthropods of the Azorean Islands. (PDF) [file pone.0033995.s004.pdf]

Supporting Information S4 Correlation (Spearman rank coefficient) between measures of rarity for 178 arthropods of the Azorean Islands.

	**NBIO**	***H'***	**Abundance**	**Kattan Index with SIEs**	**Kattan index with AZEs**
**NISL**	0.610	0.472	0.686	-0.742	-0.515
**NBIO**		0.887	0.559	-0.806	-0.708
H'			0.359	-0.742	-0.699
**Abundance**				-0.550	-0.370
**Kattan Index with SIEs**					0.787

H': Shannon index of species abundances across habitat; NISL: number of islands from which a species is known; NBIO: number of biotopes occupied by a species; SIEs: single island endemics; AZEs: Azorean endemics. All correlations are significant at $P<0.001$.
