# Supplementary material for: Use of Arthropod Rarity for Area Prioritisation: Insights from the Azorean Islands
Source: PLoS One. 2012 Mar 30;7(3):e33995. doi: 10.1371/journal.pone.0033995 (PMC3316514; doi:10.1371/journal.pone.0033995)
Supplement: Information S5 — Correlation (Spearman rank coefficient) between Index of Biodiversity Conservation Concern and Index of Biodiversity Conservation Weight for 18 Azorean forest fragments. (PDF) [file pone.0033995.s005.pdf]

Supporting Information S5 Correlation (Spearman rank coefficient) between Index of Biodiversity Conservation Concern and Index of Biodiversity Conservation Weight for 18 Azorean forest fragments.

	**BCC with AZEs**	**BCW with SIEs**	**BCW with AZEs**
**BCC with SIEs**	0.583*	0.814***	0.595**
**BCC with AZEs**		0.383	0.428
**BCW with SIEs**			0.911***

BCC: Index of Biodiversity Conservation Concern; BCW: Index of Biodiversity Conservation Weight. BCC and BCW were calculated using single island endemics (SIEs) and Azorean endemics (AZEs).
* P<0.05, ** P<0.01, *** P<0.001.
