# Supplementary material for: Use of Arthropod Rarity for Area Prioritisation: Insights from the Azorean Islands
Source: PLoS One. 2012 Mar 30;7(3):e33995. doi: 10.1371/journal.pone.0033995 (PMC3316514; doi:10.1371/journal.pone.0033995)
Supplement: Information S6 — Area Under the Curve (AUC) values for the potential distribution modelling of all 47 studied species. (PDF) [file pone.0033995.s006.pdf]

Supporting Information S6 Area Under the Curve (AUC) values for the potential distribution modelling of all 47 studied species.

**Species**	**AUC**	**Species**	**AUC**
Acorigone acoreensis	0.990	*Lithobius pilicornis pilicornis*	0.881
Acyrthosiphon pisum	0.861	*Megamelodes quadrimaculatus*	0.910
Alestrus dolosus	0.997	*Mythimna unipuncta*	0.717
Anaspis proteus	0.903	*Nabis pseudoferus ibericus*	0.743
Anoscopus albifrons	0.832	*Neon acoreensis*	0.997
Aphrodes hamiltoni	0.976	*Ocypus aethiops*	0.942
Argyresthia atlanticella	0.935	*Ocypus olens*	0.750
Astenus lyonessius	0.766	*Palliduphantes schmitzi*	0.939
Calosoma olivieri	0.781	*Pardosa acorensis*	0.775
Catops coracinus	0.857	*Pisaura acoreensis*	0.994
Cedrorum azoricus azoricus	0.990	*Porrhomma borgesi*	0.978
Cixius azoterceirae	0.951	*Pseudoplectus perplexus*	0.925
Cryptops hortensis	0.966	*Psylliodes marcidus*	0.790
Drouetius azoricus parallelirostris	0.985	*Quedius simplicifrons*	0.952
Drouetius borgesi borgesi	0.975	*Rugathodes acoreensis*	0.983
Emblethis denticollis	0.882	*Rugilus orbiculatus orbiculatus*	0.810
Euscelidius variegatus	0.816	*Sancus acoreensis*	0.974
Geophilus truncorum	0.936	*Scolopostethus decoratus*	0.834
Geotomus punctulatus	0.881	*Scoparia coecimaculalis*	0.931
Heteroderes azoricus	0.772	*Tenuiphantes miguelensis*	0.927
Hirticollis quadriguttatus	0.763	*Trechus terrabravensis*	0.991
Homalenotus coriaceus	0.846	*Trigoniophthalmus borgesi*	0.951
Leiobunum blackwalli	0.858	*Zetha vestita*	0.826
Lepthyphantes acoreensis	0.948		
